# Dexmedetomidine Used to Maintain Spontaneous Ventilation in a Patient With Anterior Mediastinal Mass and Superior Vena Cava Syndrome

**DOI:** 10.31486/toj.23.0055

**Published:** 2023

**Authors:** Daniel J. Olix, Dominic S. Carollo, Kelly G. Ural

**Affiliations:** ^1^ Department of Anesthesiology and Perioperative Medicine, Ochsner Clinic Foundation, New Orleans, LA; ^2^ The University of Queensland Medical School, Ochsner Clinical School, New Orleans, LA

**Keywords:** *Dexmedetomidine*, *mediastinal disease*, *superior vena cava syndrome*

## Abstract

**Background:** Anterior mediastinal masses (AMMs), which can be benign or malignant, are a common cause of superior vena cava (SVC) syndrome. Because of their location, AMMs can cause significant airway compromise during the perioperative period, so anesthetic management of a patient with SVC syndrome can present significant challenges.

**Case Report:** A patient presented with SVC syndrome secondary to a large AMM. After careful consideration and discussion with the patient about the risks and benefits of various approaches, the decision was made to provide sedation using dexmedetomidine as the sole agent during image-guided biopsy.

**Conclusion:** Patients who present with AMMs require careful anesthetic planning. Dexmedetomidine can be effective in achieving the primary objective of maintaining spontaneous respiration.

## INTRODUCTION

The term superior vena cava (SVC) syndrome is used to describe a collection of symptoms in patients who have SVC compression and resultant venous congestion leading to face and neck swelling.^1^ Anterior mediastinal masses (AMMs) are a common cause of SVC syndrome. AMMs can be benign or malignant and include thymomas, thyroid carcinomas, lymphomas, and mixed germ cell tumors. While many of these tumors require surgical resection, others may respond well to chemotherapy and radiation. Obtaining a quality tissue biopsy is essential in guiding the therapy.

Because of their location, AMMs can cause significant airway compromise during the perioperative period. The anesthetic approach used during tissue biopsy must be carefully planned, as general anesthesia can exacerbate the effects of airway and vascular compression.^2^ We present the case of a patient who had difficulty lying flat, shortness of breath with exertion, and extreme anxiety. After careful consideration and discussion with the patient about the risks and benefits of various approaches, the decision was made to provide biopsy sedation using dexmedetomidine as the sole agent.

Written informed consent was obtained, including authorization to use photography and written Health Insurance Portability and Accountability Act authorization for the publication of this case report. The CARE checklist was used to facilitate the writing of this manuscript.

## CASE REPORT

**Figure 1.**
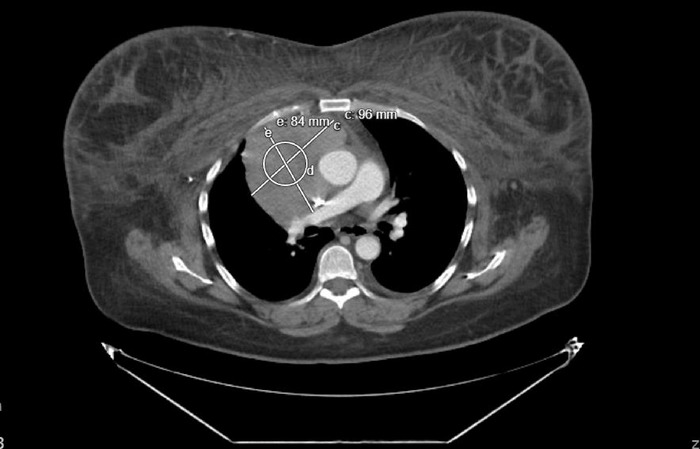
Initial computed tomography scan showing a large anterior mediastinal mass causing superior vena cava compression.

A 49-year-old female with no significant medical history first presented to urgent care for face, neck, and breast swelling. She was given a prednisolone injection without improvement. Chest x-ray showed mediastinal widening, and chest computed tomography (CT) scan with contrast revealed an AMM that measured 9.6 × 8.4 cm with retrosternal extension, likely encasement and narrowing of the right upper and middle lobe pulmonary arterial branches, and generalized upper body wall edema (Figure 1). The patient was referred to thoracic surgery and advised to go to the emergency department (ED) if she had any difficulty breathing.

**Figure 2.**
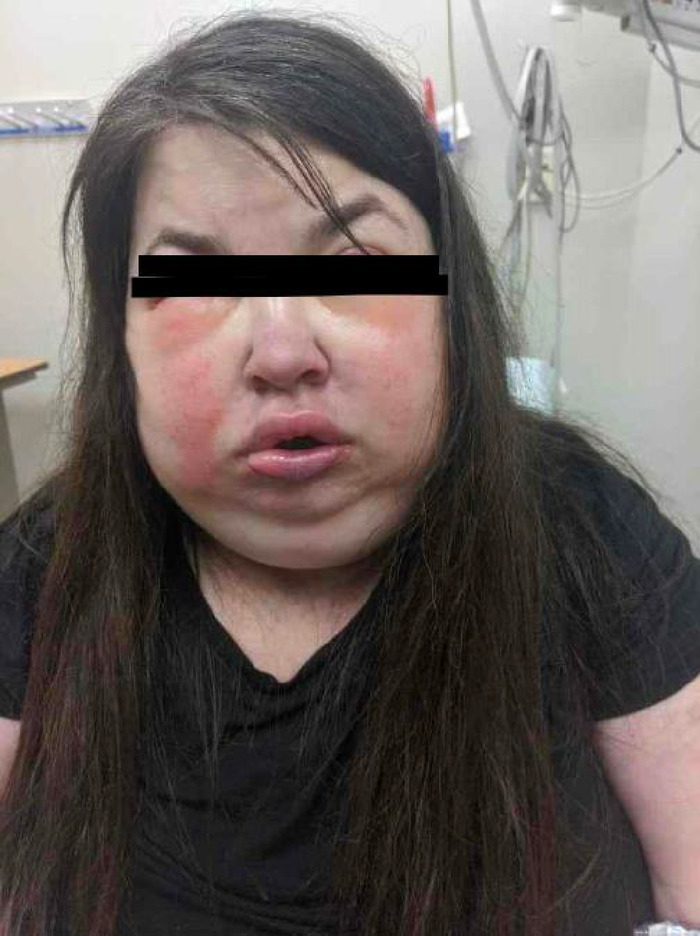
The patient had extensive face and neck swelling on presentation to the emergency department.

Two days later, thoracic surgery reviewed the scan and recommended a CT-guided biopsy with interventional radiology as the AMM was not surgically resectable. The AMM was concerning for lymphoblastic lymphoma vs aggressive thymic tumor. The same day, the patient presented to the ED with progression of her symptoms. She had not been able to tolerate anything by mouth for 2 days other than small amounts of liquid, and she complained of shortness of breath when supine and with exertion. She also had extensive face and neck swelling (Figure 2). The patient was transferred to our facility for a higher level of care, and the anesthesia team was consulted to provide sedation for an image-guided biopsy with interventional radiology.

On examination, her mouth opening was <3 cm, Mallampati score was class IV with no improvement with phonation, thyromental distance was <4 cm, and neck findings were “large, swollen, and warm.” The anesthesia preprocedure plan was for gentle sedation with dexmedetomidine. The primary goal was to preserve the patient's spontaneous ventilation while allowing interventional radiology to obtain a good tissue sample.

The patient was administered 1 mg midazolam, then 80 μg of dexmedetomidine was titrated over 10 minutes for induction, and then a dexmedetomidine infusion was continued at 1.5 μg/kg/hr for the duration of the procedure. Vital signs remained stable throughout the procedure, with blood pressures of 149 to 168/94 to 99 mm Hg and heart rate of 75 to 126 beats/min. Oxygen saturation was maintained at 95% to 98% on a 3 L face mask at 100% fraction of inspired oxygen.

After the procedure, the patient was transported to the postanesthesia care unit on 6 to 10 L/min oxygen by face mask with spontaneous ventilation. She had no apparent anesthetic complications. The biopsy pathology report revealed primary mediastinal (thymic) large B-cell lymphoma.

The next day, a 5 French peripherally inserted central catheter was placed with ultrasound guidance for initiation of DA-EPOCH chemotherapy regimen (doxorubicin 20 mg, etoposide phosphate 102 mg, vincristine sulfate 0.8 mg). The patient tolerated cycle 1 of her chemotherapy well and was discharged from the hospital 7 days after her initial presentation. Four additional cycles of DA-EPOCH treatments were administered in the outpatient infusion clinic, as well as Udenyca (pegfilgastrim-cbqv) and Rituxan (rituximab) infusions with a positive therapeutic response. Her most recent positron emission tomography scan, 1 year after initial diagnosis and treatment, showed no evidence of disease recurrence.

## DISCUSSION

### Superior Vena Cava Syndrome

SVC syndrome is caused by impairment of blood flow through the SVC to the right atrium of the heart and can be a life-threatening condition associated with high morbidity and mortality. Common causes can be malignant or benign in nature, with malignant causes most commonly from thyroid cancer, mixed germ cell tumors, or lymphomas, and benign causes most commonly from thymomas or other thymic tumors, nonmalignant thyroid tumors, or cystic hygromas.^2^ Common signs of SVC syndrome include dyspnea, facial and neck swelling, venous distention evident in the neck or chest wall, stridor, and cough. These symptoms are often worse when the patient is leaning forward, coughing, or lying down.^3^

### Anesthesia Considerations in Superior Vena Cava Syndrome

For the anesthesiologist, SVC syndrome can cause considerable intraoperative challenges. SVC syndrome caused by an AMM can lead to severe and often life-threatening complications because of the compression of major airways and vascular structures.^2^ The airway complications can be exacerbated by general anesthesia and are not always alleviated by intubation. Up to 20% of patients with AMM undergoing general anesthesia have been reported to experience serious complications.^4^

Complication rates increase with a decrease in tracheal cross-sectional area, compression of either mainstem bronchus, or a pericardial effusion.^4^ Thorough preoperative evaluation is highly recommended. The most concerning symptoms that indicate airway compromise are tracheal compression, orthopnea, and stridor.^5^ Preprocedure imaging with either a thoracic CT scan or a lateral view chest x-ray may be extremely useful in risk stratification, as a reduction in tracheal cross-sectional area >50% is associated with an increased risk of mortality.^3,6^

Preoperative tracheal compression is one of the highest risk factors for intraoperative anesthetic complications, as general anesthesia exacerbates the compression by reducing lung volumes and relaxing bronchial smooth muscle.^2,6^ Functional residual capacity is reduced by an estimated 20% under general anesthesia.^4^ General anesthesia, in combination with loss of spontaneous diaphragmatic activity due to paralysis and intermittent positive pressure ventilation, reduces transpleural pressure gradients that in turn reduce the caliber of large airways, worsening compression and leading to airway collapse.^6,7^

Several recommendations have been made for intraoperative management of patients with SVC syndrome. One of the most common is to maintain spontaneous ventilation throughout the case and avoid paralytics when possible.^4,8^ Another recommendation is to use local anesthesia, but if general anesthesia cannot be avoided in high-risk patients, preparing for cardiopulmonary bypass after induction is recommended.^4,9^ A plan should also be in place for rapid resuscitation that includes lower extremity vascular access, as upper extremity access may be compromised by SVC syndrome.^3^ In patients with large AMMs, upper extremity access may increase the risk of hemorrhage, embolization, incorrect blood pressure readings, and unpredictable drug effects.^10^ One of the most critical concerns for anesthetic management of patients with severe SVC syndrome undergoing general anesthesia is prolonged intubation if the obstruction from SVC syndrome cannot be relieved. Additionally, the longer the patient is under general anesthesia with prolonged compression of the trachea, the higher the risk of tracheomalacia that can further complicate plans for extubation.^10^

### Dexmedetomidine

The US Food and Drug Administration approved dexmedetomidine in the late 1990s for sedation of intubated patients in critical care settings and for sedation of nonintubated patients undergoing surgery or other procedures.^4^ Dexmedetomidine is a highly selective alpha-2 agonist that produces sedation, anxiolysis, and analgesia and can facilitate rapid and smooth awakening. Importantly, dexmedetomidine does not cause respiratory depression even at high dosages.^9^

Most of the literature regarding the use of dexmedetomidine for general anesthesia is in the pediatric population, with few case reports in adults. Nafiu et al describe using dexmedetomidine for a patient with severe needle phobia undergoing a Chamberlain procedure for a large mediastinal mass with significant airway compression.^9^ The patient did not tolerate the procedure under local anesthesia, but the biopsy was successfully completed using dexmedetomidine for sedation. Abdelmalak et al describe using dexmedetomidine at sedative doses in 2 patients with AMM who presented for laser tumor debulking and rigid bronchoscopy for stent placement.^11^ We have described the successful anesthetic management of a patient undergoing an image-guided biopsy of an AMM with dexmedetomidine used as the sole anesthetic agent to maintain airway integrity.

Given its anesthetic and analgesic properties, dexmedetomidine has many benefits compared to other agents. It aids in maintenance of spontaneous ventilation even at relatively high doses while producing an amnestic state with loss of awareness of the external environment.^4,11^ Additionally, dexmedetomidine has analgesic properties without the neuropsychiatric effects of ketamine.^9^ Notably, dose-dependent bradycardia and hypotension can occur at higher doses of dexmedetomidine, and increased systematic and pulmonary vascular resistance has been described with rapid infusion.^9,11^ However, in adults, infusion doses up to 10 μg/kg/hr have been reported without apnea or airway obstruction.^4^

## CONCLUSION

Patients presenting with AMMs require careful anesthetic planning, and dexmedetomidine can be effective in maintaining spontaneous respiration.

## Notes

*This article meets the Accreditation Council for Graduate Medical Education and the American Board of Medical Specialties Maintenance of Certification competencies for Patient Care and Medical Knowledge.*
